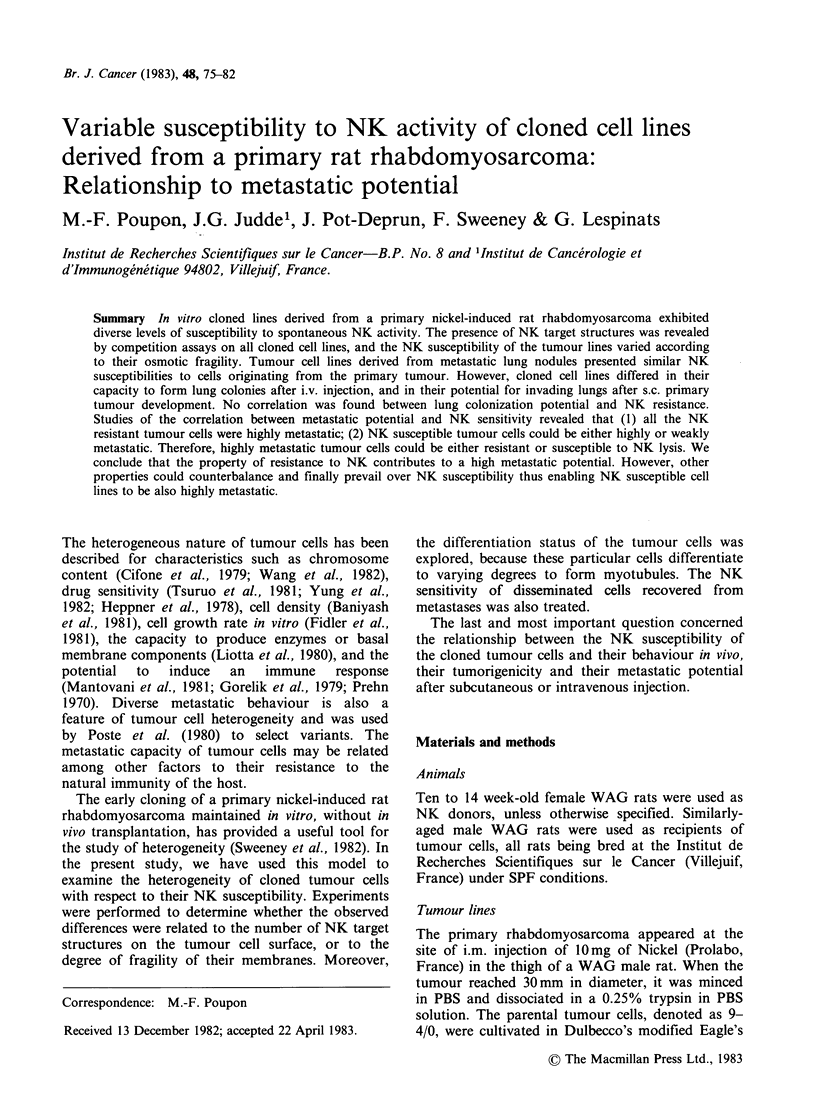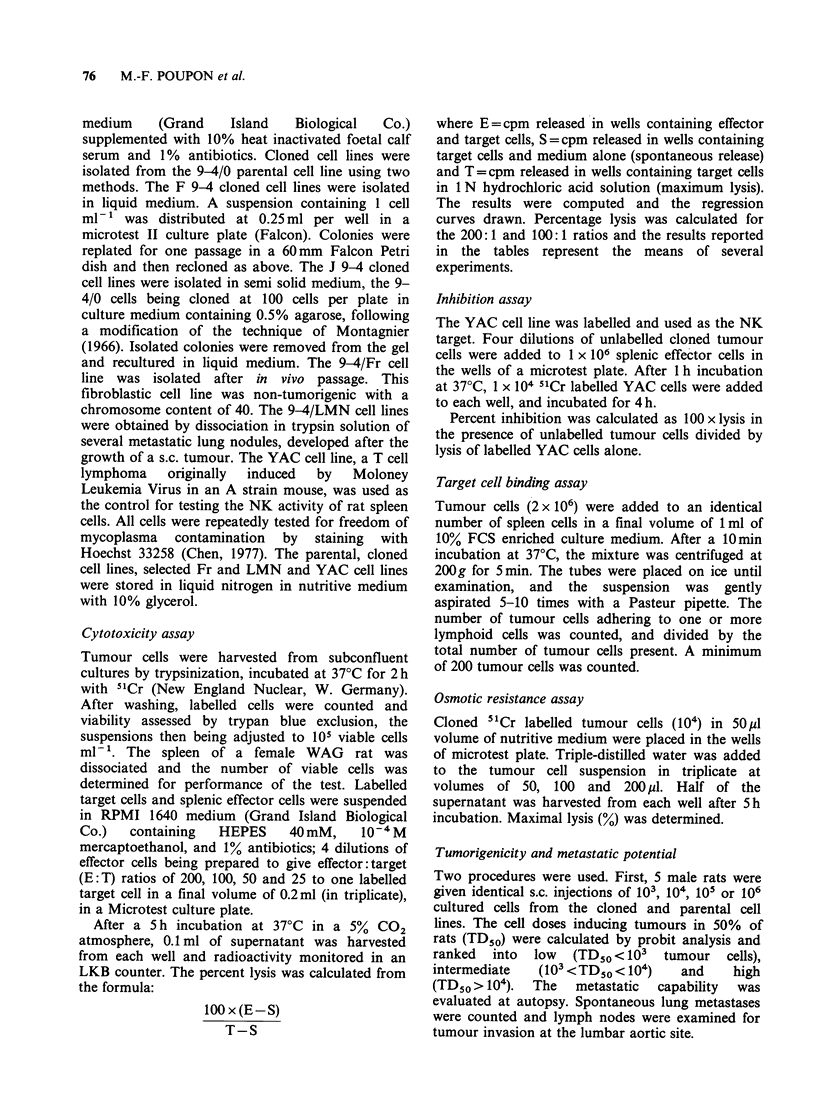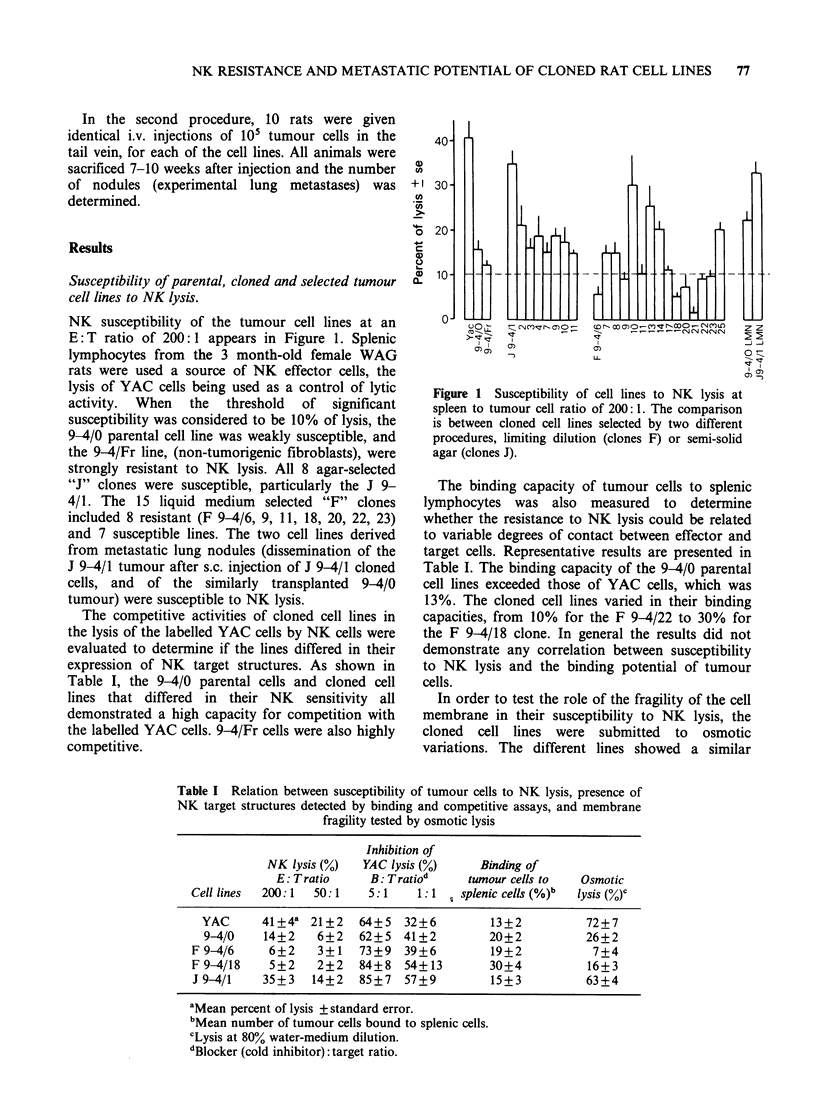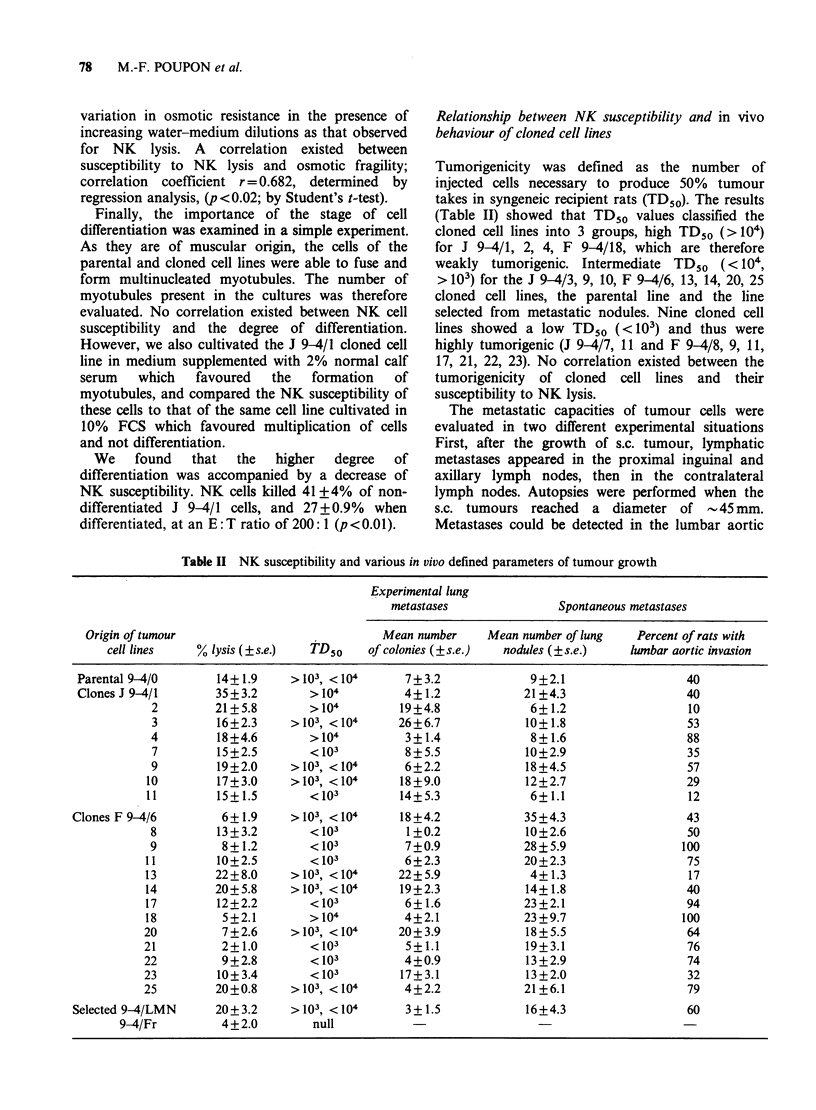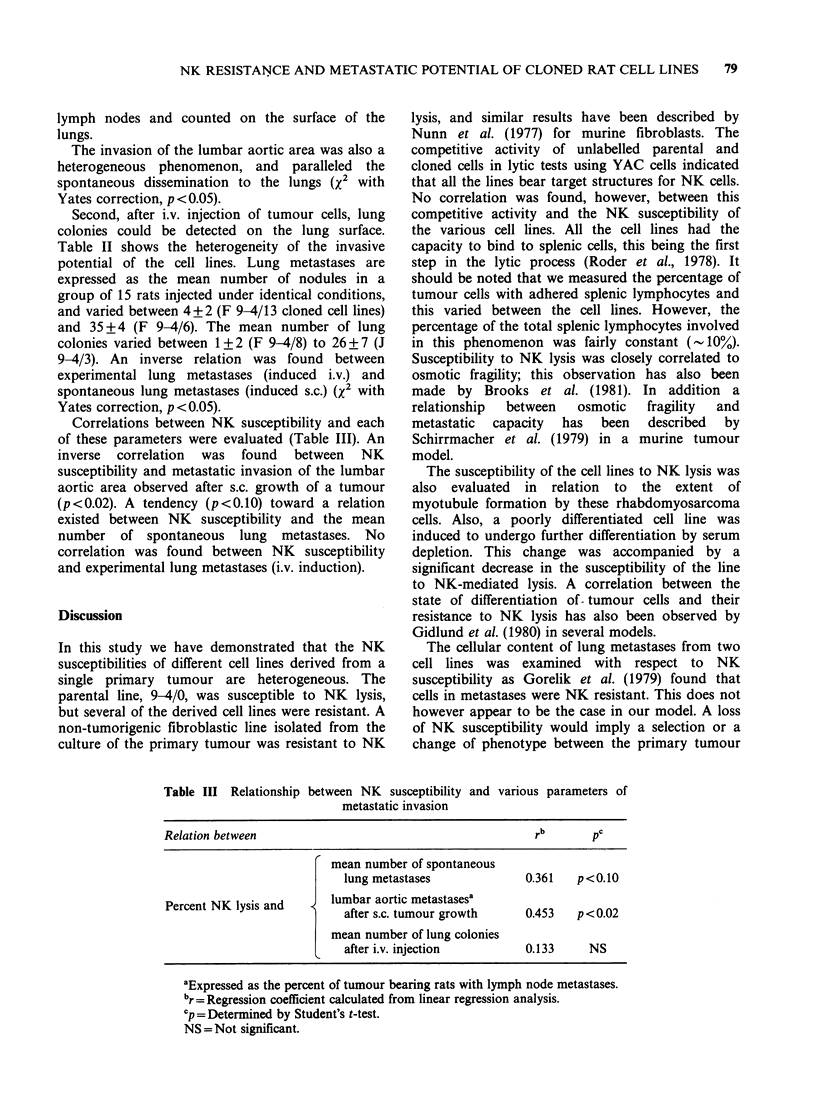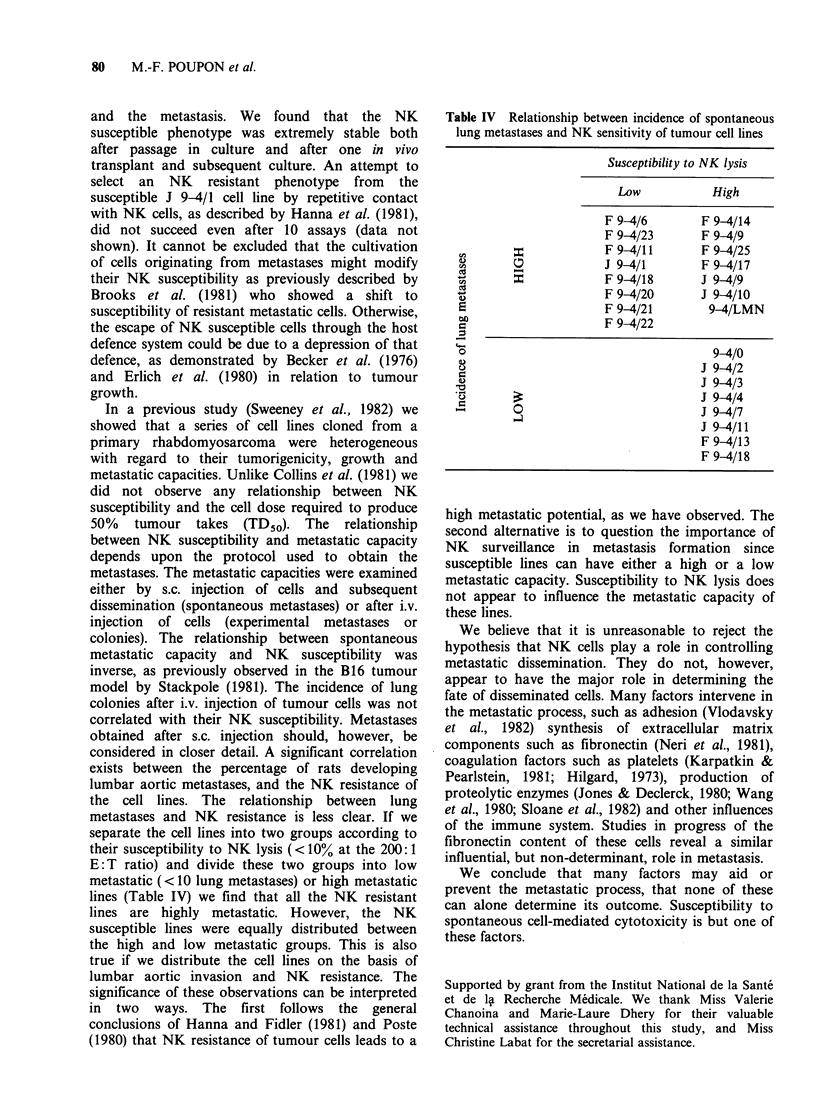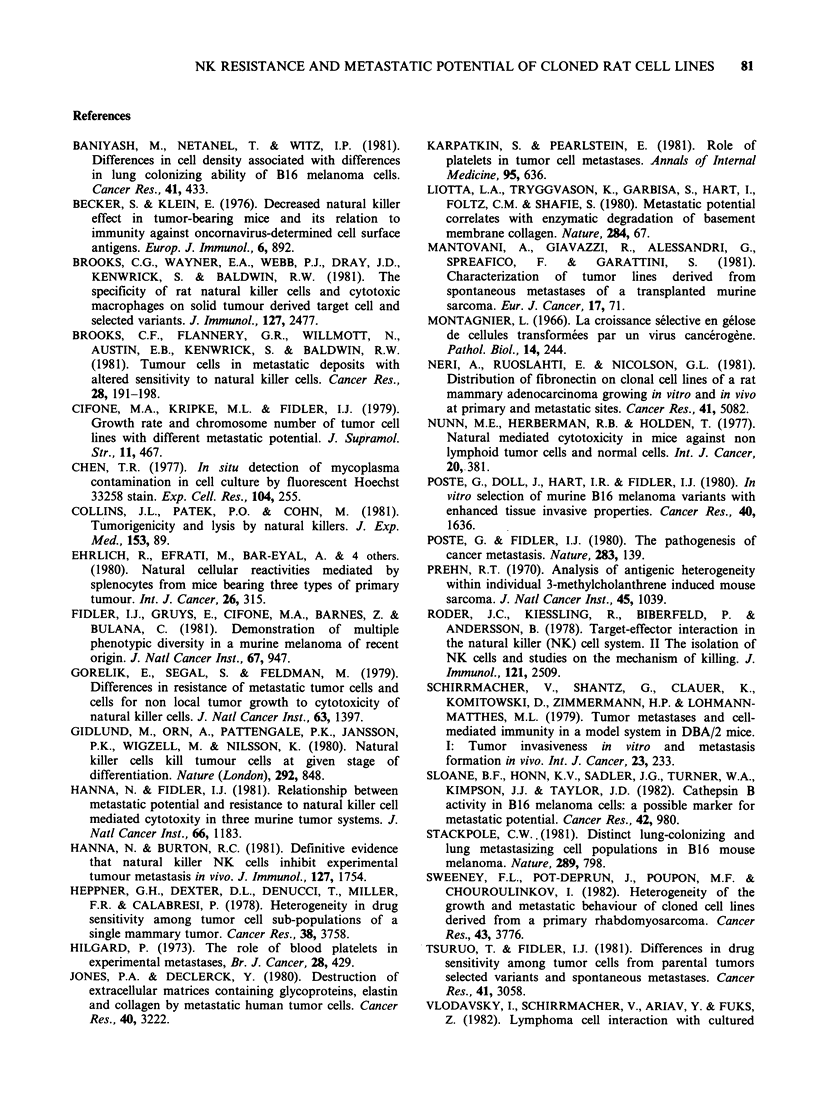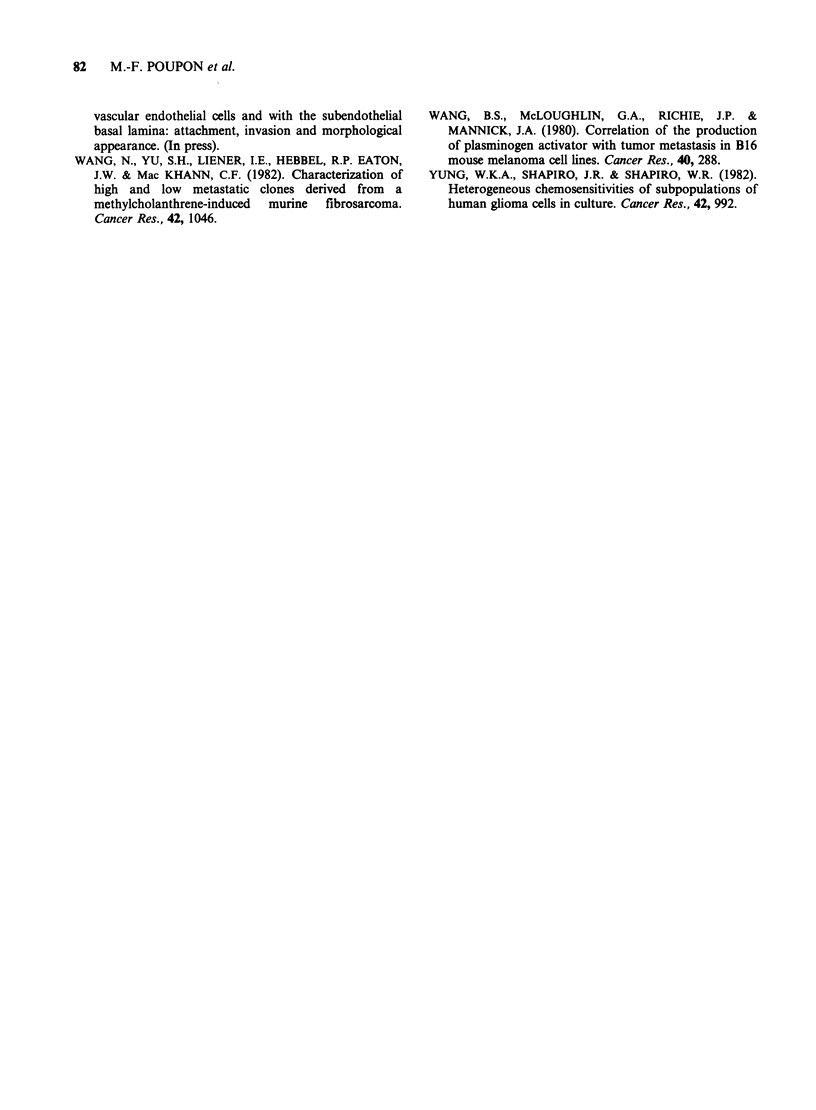# Variable susceptibility to NK activity of cloned cell lines derived from a primary rat rhabdomyosarcoma: relationship to metastatic potential.

**DOI:** 10.1038/bjc.1983.159

**Published:** 1983-07

**Authors:** M. F. Poupon, J. G. Judde, J. Pot-Deprun, F. Sweeney, G. Lespinats

## Abstract

In vitro cloned lines derived from a primary nickel-induced rat rhabdomyosarcoma exhibited diverse levels of susceptibility to spontaneous NK activity. The presence of NK target structures was revealed by competition assays on all cloned cell lines, and the NK susceptibility of the tumour lines varied according to their osmotic fragility. Tumour cell lines derived from metastatic lung nodules presented similar NK susceptibilities to cells originating from the primary tumour. However, cloned cell lines differed in their capacity to form lung colonies after i.v. injection, and in their potential for invading lungs after s.c. primary tumour development. No correlation was found between lung colonization potential and NK resistance. Studies of the correlation between metastatic potential and NK sensitivity revealed that (1) all the NK resistant tumour cells were highly metastatic; (2) NK susceptible tumour cells could be either highly or weakly metastatic. Therefore, highly metastatic tumour cells could be either resistant or susceptible to NK lysis. We conclude that the property of resistance to NK contributes to a high metastatic potential. However, other properties could counterbalance and finally prevail over NK susceptibility thus enabling NK susceptible cell lines to be also highly metastatic.


*Br. J. Cancer* (1983), **48**, 75–82

### Variable susceptibility to NK activity of cloned cell lines derived from a primary rat rhabdomyosarcoma: Relationship to metastatic potential

M.-F. Poupon, J.G. Judde[1], J. Pot-Deprun, F. Sweeney & G. Lespinats

*Institut de Recherches Scientifiques sur le Cancer—B.P. No. 8 and [1]Institut de Cancérologie et d'Immunogénétique 94802, Villejuif, France.*

**Summary** *In vitro* cloned lines derived from a primary nickel-induced rat rhabdomyosarcoma exhibited diverse levels of susceptibility to spontaneous NK activity. The presence of NK target structures was revealed by competition assays on all cloned cell lines, and the NK susceptibility of the tumour lines varied according to their osmotic fragility. Tumour cell lines derived from metastatic lung nodules presented similar NK susceptibilities to cells originating from the primary tumour. However, cloned cell lines differed in their capacity to form lung colonies after i.v. injection, and in their potential for invading lungs after s.c. primary tumour development. No correlation was found between lung colonization potential and NK resistance. Studies of the correlation between metastatic potential and NK sensitivity revealed that (1) all the NK resistant tumour cells were highly metastatic; (2) NK susceptible tumour cells could be either highly or weakly metastatic. Therefore, highly metastatic tumour cells could be either resistant or susceptible to NK lysis. We conclude that the property of resistance to NK contributes to a high metastatic potential. However, other properties could counterbalance and finally prevail over NK susceptibility thus enabling NK susceptible cell lines to be also highly metastatic.

The heterogeneous nature of tumour cells has been described for characteristics such as chromosome content (Cifone *et al.*, 1979; Wang *et al.*, 1982), drug sensitivity (Tsuruo *et al.*, 1981; Yung *et al.*, 1982; Heppner *et al.*, 1978), cell density (Baniyash *et al.*, 1981), cell growth rate *in vitro* (Fidler *et al.*, 1981), the capacity to produce enzymes or basal membrane components (Liotta *et al.*, 1980), and the potential to induce an immune response (Mantovani *et al.*, 1981; Gorelik *et al.*, 1979; Prehn 1970). Diverse metastatic behaviour is also a feature of tumour cell heterogeneity and was used by Poste *et al.* (1980) to select variants. The metastatic capacity of tumour cells may be related among other factors to their resistance to the natural immunity of the host.

The early cloning of a primary nickel-induced rat rhabdomyosarcoma maintained *in vitro*, without *in vivo* transplantation, has provided a useful tool for the study of heterogeneity (Sweeney *et al.*, 1982). In the present study, we have used this model to examine the heterogeneity of cloned tumour cells with respect to their NK susceptibility. Experiments were performed to determine whether the observed differences were related to the number of NK target structures on the tumour cell surface, or to the degree of fragility of their membranes. Moreover, the differentiation status of the tumour cells was explored, because these particular cells differentiate to varying degrees to form myotubules. The NK sensitivity of disseminated cells recovered from metastases was also treated.

The last and most important question concerned the relationship between the NK susceptibility of the cloned tumour cells and their behaviour *in vivo*, their tumorigenicity and their metastatic potential after subcutaneous or intravenous injection.

### Materials and methods

### *Animals*

Ten to 14 week-old female WAG rats were used as NK donors, unless otherwise specified. Similarly-aged male WAG rats were used as recipients of tumour cells, all rats being bred at the Institut de Recherches Scientifiques sur le Cancer (Villejuif, France) under SPF conditions.

### *Tumour lines*

The primary rhabdomyosarcoma appeared at the site of i.m. injection of 10 mg of Nickel (Prolabo, France) in the thigh of a WAG male rat. When the tumour reached 30 mm in diameter, it was minced in PBS and dissociated in a 0.25% trypsin in PBS solution. The parental tumour cells, denoted as 9-4/0, were cultivated in Dulbecco's modified Eagle's

Correspondence: M.-F. Poupon

Received 13 December 1982; accepted 22 April 1983.

© The Macmillan Press Ltd., 1983

medium (Grand Island Biological Co.) supplemented with 10% heat inactivated foetal calf serum and 1% antibiotics. Cloned cell lines were isolated from the 9–4/0 parental cell line using two methods. The F 9–4 cloned cell lines were isolated in liquid medium. A suspension containing 1 cell $ml^{-1}$ was distributed at 0.25 ml per well in a microtest II culture plate (Falcon). Colonies were replated for one passage in a 60 mm Falcon Petri dish and then recloned as above. The J 9–4 cloned cell lines were isolated in semi solid medium, the 9–4/0 cells being cloned at 100 cells per plate in culture medium containing 0.5% agarose, following a modification of the technique of Montagnier (1966). Isolated colonies were removed from the gel and recultured in liquid medium. The 9–4/Fr cell line was isolated after *in vivo* passage. This fibroblastic cell line was non-tumorigenic with a chromosome content of 40. The 9–4/LMN cell lines were obtained by dissociation in trypsin solution of several metastatic lung nodules, developed after the growth of a s.c. tumour. The YAC cell line, a T cell lymphoma originally induced by Moloney Leukemia Virus in an A strain mouse, was used as the control for testing the NK activity of rat spleen cells. All cells were repeatedly tested for freedom of mycoplasma contamination by staining with Hoechst 33258 (Chen, 1977). The parental, cloned cell lines, selected Fr and LMN and YAC cell lines were stored in liquid nitrogen in nutritive medium with 10% glycerol.

*Cytotoxicity assay*

Tumour cells were harvested from subconfluent cultures by trypsinization, incubated at 37°C for 2 h with $^{51}Cr$ (New England Nuclear, W. Germany). After washing, labelled cells were counted and viability assessed by trypan blue exclusion, the suspensions then being adjusted to $10^5$ viable cells $ml^{-1}$. The spleen of a female WAG rat was dissociated and the number of viable cells was determined for performance of the test. Labelled target cells and splenic effector cells were suspended in RPMI 1640 medium (Grand Island Biological Co.) containing HEPES 40 mM, $10^{-4}$ M mercaptoethanol, and 1% antibiotics; 4 dilutions of effector cells being prepared to give effector:target (E:T) ratios of 200, 100, 50 and 25 to one labelled target cell in a final volume of 0.2 ml (in triplicate), in a Microtest culture plate.

After a 5 h incubation at 37°C in a 5% $CO_2$ atmosphere, 0.1 ml of supernatant was harvested from each well and radioactivity monitored in an LKB counter. The percent lysis was calculated from the formula:

$$\frac{100 \times (E-S)}{T-S}$$

where E = cpm released in wells containing effector and target cells, S = cpm released in wells containing target cells and medium alone (spontaneous release) and T = cpm released in wells containing target cells in 1 N hydrochloric acid solution (maximum lysis). The results were computed and the regression curves drawn. Percentage lysis was calculated for the 200:1 and 100:1 ratios and the results reported in the tables represent the means of several experiments.

*Inhibition assay*

The YAC cell line was labelled and used as the NK target. Four dilutions of unlabelled cloned tumour cells were added to $1 \times 10^6$ splenic effector cells in the wells of a microtest plate. After 1 h incubation at 37°C, $1 \times 10^4$ $^{51}Cr$ labelled YAC cells were added to each well, and incubated for 4 h.

Percent inhibition was calculated as 100 × lysis in the presence of unlabelled tumour cells divided by lysis of labelled YAC cells alone.

*Target cell binding assay*

Tumour cells ($2 \times 10^6$) were added to an identical number of spleen cells in a final volume of 1 ml of 10% FCS enriched culture medium. After a 10 min incubation at 37°C, the mixture was centrifuged at 200 *g* for 5 min. The tubes were placed on ice until examination, and the suspension was gently aspirated 5–10 times with a Pasteur pipette. The number of tumour cells adhering to one or more lymphoid cells was counted, and divided by the total number of tumour cells present. A minimum of 200 tumour cells was counted.

*Osmotic resistance assay*

Cloned $^{51}Cr$ labelled tumour cells ($10^4$) in 50 $\mu$l volume of nutritive medium were placed in the wells of microtest plate. Triple-distilled water was added to the tumour cell suspension in triplicate at volumes of 50, 100 and 200 $\mu$l. Half of the supernatant was harvested from each well after 5 h incubation. Maximal lysis (%) was determined.

*Tumorigenicity and metastatic potential*

Two procedures were used. First, 5 male rats were given identical s.c. injections of $10^3$, $10^4$, $10^5$ or $10^6$ cultured cells from the cloned and parental cell lines. The cell doses inducing tumours in 50% of rats ($TD_{50}$) were calculated by probit analysis and ranked into low ($TD_{50} < 10^3$ tumour cells), intermediate ($10^3 < TD_{50} < 10^4$) and high ($TD_{50} > 10^4$). The metastatic capability was evaluated at autopsy. Spontaneous lung metastases were counted and lymph nodes were examined for tumour invasion at the lumbar aortic site.

In the second procedure, 10 rats were given identical i.v. injections of $10^5$ tumour cells in the tail vein, for each of the cell lines. All animals were sacrificed 7–10 weeks after injection and the number of nodules (experimental lung metastases) was determined.

### Results

*Susceptibility of parental, cloned and selected tumour cell lines to NK lysis.*

NK susceptibility of the tumour cell lines at an E:T ratio of 200:1 appears in Figure 1. Splenic lymphocytes from the 3 month-old female WAG rats were used a source of NK effector cells, the lysis of YAC cells being used as a control of lytic activity. When the threshold of significant susceptibility was considered to be 10% of lysis, the 9–4/0 parental cell line was weakly susceptible, and the 9–4/Fr line, (non-tumorigenic fibroblasts), were strongly resistant to NK lysis. All 8 agar-selected "J" clones were susceptible, particularly the J 9–4/1. The 15 liquid medium selected "F" clones included 8 resistant (F 9–4/6, 9, 11, 18, 20, 22, 23) and 7 susceptible lines. The two cell lines derived from metastatic lung nodules (dissemination of the J 9–4/1 tumour after s.c. injection of J 9–4/1 cloned cells, and of the similarly transplanted 9–4/0 tumour) were susceptible to NK lysis.

The competitive activities of cloned cell lines in the lysis of the labelled YAC cells by NK cells were evaluated to determine if the lines differed in their expression of NK target structures. As shown in Table I, the 9–4/0 parental cells and cloned cell lines that differed in their NK sensitivity all demonstrated a high capacity for competition with the labelled YAC cells. 9–4/Fr cells were also highly competitive.

**Figure 1** Susceptibility of cell lines to NK lysis at spleen to tumour cell ratio of 200:1. The comparison is between cloned cell lines selected by two different procedures, limiting dilution (clones F) or semi-solid agar (clones J).

The binding capacity of tumour cells to splenic lymphocytes was also measured to determine whether the resistance to NK lysis could be related to variable degrees of contact between effector and target cells. Representative results are presented in Table I. The binding capacity of the 9–4/0 parental cell lines exceeded those of YAC cells, which was 13%. The cloned cell lines varied in their binding capacities, from 10% for the F 9–4/22 to 30% for the F 9–4/18 clone. In general the results did not demonstrate any correlation between susceptibility to NK lysis and the binding potential of tumour cells.

In order to test the role of the fragility of the cell membrane in their susceptibility to NK lysis, the cloned cell lines were submitted to osmotic variations. The different lines showed a similar

**Table I** Relation between susceptibility of tumour cells to NK lysis, presence of NK target structures detected by binding and competitive assays, and membrane fragility tested by osmotic lysis

| Cell lines | *NK lysis (%) E:T ratio* 200:1 | 50:1 | *Inhibition of YAC lysis (%) B:T ratio*[d] 5:1 | 1:1 | *Binding of tumour cells to splenic cells (%)*[b] | *Osmotic lysis (%)*[c] |
|---|---|---|---|---|---|---|
| YAC | 41±4[a] | 21±2 | 64±5 | 32±6 | 13±2 | 72±7 |
| 9–4/0 | 14±2 | 6±2 | 62±5 | 41±2 | 20±2 | 26±2 |
| F 9–4/6 | 6±2 | 3±1 | 73±9 | 39±6 | 19±2 | 7±4 |
| F 9–4/18 | 5±2 | 2±2 | 84±8 | 54±13 | 30±4 | 16±3 |
| J 9–4/1 | 35±3 | 14±2 | 85±7 | 57±9 | 15±3 | 63±4 |

[a]Mean percent of lysis ±standard error.
[b]Mean number of tumour cells bound to splenic cells.
[c]Lysis at 80% water-medium dilution.
[d]Blocker (cold inhibitor):target ratio.

variation in osmotic resistance in the presence of increasing water–medium dilutions as that observed for NK lysis. A correlation existed between susceptibility to NK lysis and osmotic fragility; correlation coefficient $r = 0.682$, determined by regression analysis, ($p < 0.02$; by Student's $t$-test).

Finally, the importance of the stage of cell differentiation was examined in a simple experiment. As they are of muscular origin, the cells of the parental and cloned cell lines were able to fuse and form multinucleated myotubules. The number of myotubules present in the cultures was therefore evaluated. No correlation existed between NK cell susceptibility and the degree of differentiation. However, we also cultivated the J 9–4/1 cloned cell line in medium supplemented with 2% normal calf serum which favoured the formation of myotubules, and compared the NK susceptibility of these cells to that of the same cell line cultivated in 10% FCS which favoured multiplication of cells and not differentiation.

We found that the higher degree of differentiation was accompanied by a decrease of NK susceptibility. NK cells killed $41 \pm 4\%$ of non-differentiated J 9–4/1 cells, and $27 \pm 0.9\%$ when differentiated, at an E:T ratio of 200:1 ($p < 0.01$).

*Relationship between NK susceptibility and* in vivo *behaviour of cloned cell lines*

Tumorigenicity was defined as the number of injected cells necessary to produce 50% tumour takes in syngeneic recipient rats ($TD_{50}$). The results (Table II) showed that $TD_{50}$ values classified the cloned cell lines into 3 groups, high $TD_{50}$ ($> 10^4$) for J 9–4/1, 2, 4, F 9–4/18, which are therefore weakly tumorigenic. Intermediate $TD_{50}$ ($< 10^4$, $> 10^3$) for the J 9–4/3, 9, 10, F 9–4/6, 13, 14, 20, 25 cloned cell lines, the parental line and the line selected from metastatic nodules. Nine cloned cell lines showed a low $TD_{50}$ ($< 10^3$) and thus were highly tumorigenic (J 9–4/7, 11 and F 9–4/8, 9, 11, 17, 21, 22, 23). No correlation existed between the tumorigenicity of cloned cell lines and their susceptibility to NK lysis.

The metastatic capacities of tumour cells were evaluated in two different experimental situations First, after the growth of s.c. tumour, lymphatic metastases appeared in the proximal inguinal and axillary lymph nodes, then in the contralateral lymph nodes. Autopsies were performed when the s.c. tumours reached a diameter of ~45 mm. Metastases could be detected in the lumbar aortic

**Table II** NK susceptibility and various *in vivo* defined parameters of tumour growth

| Origin of tumour cell lines | % lysis (±s.e.) | $TD_{50}$ | Experimental lung metastases: Mean number of colonies (±s.e.) | Spontaneous metastases: Mean number of lung nodules (±s.e.) | Spontaneous metastases: Percent of rats with lumbar aortic invasion |
|---|---|---|---|---|---|
| Parental 9–4/0 | 14±1.9 | >10³, <10⁴ | 7±3.2 | 9±2.1 | 40 |
| Clones J 9–4/1 | 35±3.2 | >10⁴ | 4±1.2 | 21±4.3 | 40 |
| 2 | 21±5.8 | >10⁴ | 19±4.8 | 6±1.2 | 10 |
| 3 | 16±2.3 | >10³, <10⁴ | 26±6.7 | 10±1.8 | 53 |
| 4 | 18±4.6 | >10⁴ | 3±1.4 | 8±1.6 | 88 |
| 7 | 15±2.5 | <10³ | 8±5.5 | 10±2.9 | 35 |
| 9 | 19±2.0 | >10³, <10⁴ | 6±2.2 | 18±4.5 | 57 |
| 10 | 17±3.0 | >10³, <10⁴ | 18±9.0 | 12±2.7 | 29 |
| 11 | 15±1.5 | <10³ | 14±5.3 | 6±1.1 | 12 |
| Clones F 9–4/6 | 6±1.9 | >10³, <10⁴ | 18±4.2 | 35±4.3 | 43 |
| 8 | 13±3.2 | <10³ | 1±0.2 | 10±2.6 | 50 |
| 9 | 8±1.2 | <10³ | 7±0.9 | 28±5.9 | 100 |
| 11 | 10±2.5 | <10³ | 6±2.3 | 20±2.3 | 75 |
| 13 | 22±8.0 | >10³, <10⁴ | 22±5.9 | 4±1.3 | 17 |
| 14 | 20±5.8 | >10³, <10⁴ | 19±2.3 | 14±1.8 | 40 |
| 17 | 12±2.2 | <10³ | 6±1.6 | 23±2.1 | 94 |
| 18 | 5±2.1 | >10⁴ | 4±2.1 | 23±9.7 | 100 |
| 20 | 7±2.6 | >10³, <10⁴ | 20±3.9 | 18±5.5 | 64 |
| 21 | 2±1.0 | <10³ | 5±1.1 | 19±3.1 | 76 |
| 22 | 9±2.8 | <10³ | 4±0.9 | 13±2.9 | 74 |
| 23 | 10±3.4 | <10³ | 17±3.1 | 13±2.0 | 32 |
| 25 | 20±0.8 | >10³, <10⁴ | 4±2.2 | 21±6.1 | 79 |
| Selected 9–4/LMN | 20±3.2 | >10³, <10⁴ | 3±1.5 | 16±4.3 | 60 |
| 9–4/Fr | 4±2.0 | null | — | — | — |

lymph nodes and counted on the surface of the lungs.

The invasion of the lumbar aortic area was also a heterogeneous phenomenon, and paralleled the spontaneous dissemination to the lungs ($\chi^2$ with Yates correction, $p<0.05$).

Second, after i.v. injection of tumour cells, lung colonies could be detected on the lung surface. Table II shows the heterogeneity of the invasive potential of the cell lines. Lung metastases are expressed as the mean number of nodules in a group of 15 rats injected under identical conditions, and varied between $4 \pm 2$ (F 9–4/13 cloned cell lines) and $35 \pm 4$ (F 9–4/6). The mean number of lung colonies varied between $1 \pm 2$ (F 9–4/8) to $26 \pm 7$ (J 9–4/3). An inverse relation was found between experimental lung metastases (induced i.v.) and spontaneous lung metastases (induced s.c.) ($\chi^2$ with Yates correction, $p<0.05$).

Correlations between NK susceptibility and each of these parameters were evaluated (Table III). An inverse correlation was found between NK susceptibility and metastatic invasion of the lumbar aortic area observed after s.c. growth of a tumour ($p<0.02$). A tendency ($p<0.10$) toward a relation existed between NK susceptibility and the mean number of spontaneous lung metastases. No correlation was found between NK susceptibility and experimental lung metastases (i.v. induction).

### Discussion

In this study we have demonstrated that the NK susceptibilities of different cell lines derived from a single primary tumour are heterogeneous. The parental line, 9–4/0, was susceptible to NK lysis, but several of the derived cell lines were resistant. A non-tumorigenic fibroblastic line isolated from the culture of the primary tumour was resistant to NK lysis, and similar results have been described by Nunn *et al.* (1977) for murine fibroblasts. The competitive activity of unlabelled parental and cloned cells in lytic tests using YAC cells indicated that all the lines bear target structures for NK cells. No correlation was found, however, between this competitive activity and the NK susceptibility of the various cell lines. All the cell lines had the capacity to bind to splenic cells, this being the first step in the lytic process (Roder *et al.*, 1978). It should be noted that we measured the percentage of tumour cells with adhered splenic lymphocytes and this varied between the cell lines. However, the percentage of the total splenic lymphocytes involved in this phenomenon was fairly constant (~10%). Susceptibility to NK lysis was closely correlated to osmotic fragility; this observation has also been made by Brooks *et al.* (1981). In addition a relationship between osmotic fragility and metastatic capacity has been described by Schirrmacher *et al.* (1979) in a murine tumour model.

The susceptibility of the cell lines to NK lysis was also evaluated in relation to the extent of myotubule formation by these rhabdomyosarcoma cells. Also, a poorly differentiated cell line was induced to undergo further differentiation by serum depletion. This change was accompanied by a significant decrease in the susceptibility of the line to NK-mediated lysis. A correlation between the state of differentiation of tumour cells and their resistance to NK lysis has also been observed by Gidlund *et al.* (1980) in several models.

The cellular content of lung metastases from two cell lines was examined with respect to NK susceptibility as Gorelik *et al.* (1979) found that cells in metastases were NK resistant. This does not however appear to be the case in our model. A loss of NK susceptibility would imply a selection or a change of phenotype between the primary tumour

**Table III** Relationship between NK susceptibility and various parameters of metastatic invasion

| *Relation between* | | $r^b$ | $p^c$ |
|---|---|---|---|
| Percent NK lysis and | mean number of spontaneous lung metastases | 0.361 | $p<0.10$ |
| | lumbar aortic metastases[a] after s.c. tumour growth | 0.453 | $p<0.02$ |
| | mean number of lung colonies after i.v. injection | 0.133 | NS |

[a]Expressed as the percent of tumour bearing rats with lymph node metastases.
[b]$r$ = Regression coefficient calculated from linear regression analysis.
[c]$p$ = Determined by Student's $t$-test.
NS = Not significant.

and the metastasis. We found that the NK susceptible phenotype was extremely stable both after passage in culture and after one *in vivo* transplant and subsequent culture. An attempt to select an NK resistant phenotype from the susceptible J 9–4/1 cell line by repetitive contact with NK cells, as described by Hanna *et al.* (1981), did not succeed even after 10 assays (data not shown). It cannot be excluded that the cultivation of cells originating from metastases might modify their NK susceptibility as previously described by Brooks *et al.* (1981) who showed a shift to susceptibility of resistant metastatic cells. Otherwise, the escape of NK susceptible cells through the host defence system could be due to a depression of that defence, as demonstrated by Becker *et al.* (1976) and Erlich *et al.* (1980) in relation to tumour growth.

In a previous study (Sweeney *et al.*, 1982) we showed that a series of cell lines cloned from a primary rhabdomyosarcoma were heterogeneous with regard to their tumorigenicity, growth and metastatic capacities. Unlike Collins *et al.* (1981) we did not observe any relationship between NK susceptibility and the cell dose required to produce 50% tumour takes ($TD_{50}$). The relationship between NK susceptibility and metastatic capacity depends upon the protocol used to obtain the metastases. The metastatic capacities were examined either by s.c. injection of cells and subsequent dissemination (spontaneous metastases) or after i.v. injection of cells (experimental metastases or colonies). The relationship between spontaneous metastatic capacity and NK susceptibility was inverse, as previously observed in the B16 tumour model by Stackpole (1981). The incidence of lung colonies after i.v. injection of tumour cells was not correlated with their NK susceptibility. Metastases obtained after s.c. injection should, however, be considered in closer detail. A significant correlation exists between the percentage of rats developing lumbar aortic metastases, and the NK resistance of the cell lines. The relationship between lung metastases and NK resistance is less clear. If we separate the cell lines into two groups according to their susceptibility to NK lysis (<10% at the 200:1 E:T ratio) and divide these two groups into low metastatic (<10 lung metastases) or high metastatic lines (Table IV) we find that all the NK resistant lines are highly metastatic. However, the NK susceptible lines were equally distributed between the high and low metastatic groups. This is also true if we distribute the cell lines on the basis of lumbar aortic invasion and NK resistance. The significance of these observations can be interpreted in two ways. The first follows the general conclusions of Hanna and Fidler (1981) and Poste (1980) that NK resistance of tumour cells leads to a high metastatic potential, as we have observed. The second alternative is to question the importance of NK surveillance in metastasis formation since susceptible lines can have either a high or a low metastatic capacity. Susceptibility to NK lysis does not appear to influence the metastatic capacity of these lines.

**Table IV** Relationship between incidence of spontaneous lung metastases and NK sensitivity of tumour cell lines

| Incidence of lung metastases | | *Susceptibility to NK lysis* | |
|---|---|---|---|
| | | *Low* | *High* |
| HIGH | | F 9–4/6 | F 9–4/14 |
| | | F 9–4/23 | F 9–4/9 |
| | | F 9–4/11 | F 9–4/25 |
| | | J 9–4/1 | F 9–4/17 |
| | | F 9–4/18 | J 9–4/9 |
| | | F 9–4/20 | J 9–4/10 |
| | | F 9–4/21 | 9–4/LMN |
| | | F 9–4/22 | |
| LOW | | | 9–4/0 |
| | | | J 9–4/2 |
| | | | J 9–4/3 |
| | | | J 9–4/4 |
| | | | J 9–4/7 |
| | | | J 9–4/11 |
| | | | F 9–4/13 |
| | | | F 9–4/18 |

We believe that it is unreasonable to reject the hypothesis that NK cells play a role in controlling metastatic dissemination. They do not, however, appear to have the major role in determining the fate of disseminated cells. Many factors intervene in the metastatic process, such as adhesion (Vlodavsky *et al.*, 1982) synthesis of extracellular matrix components such as fibronectin (Neri *et al.*, 1981), coagulation factors such as platelets (Karpatkin & Pearlstein, 1981; Hilgard, 1973), production of proteolytic enzymes (Jones & Declerck, 1980; Wang *et al.*, 1980; Sloane *et al.*, 1982) and other influences of the immune system. Studies in progress of the fibronectin content of these cells reveal a similar influential, but non-determinant, role in metastasis.

We conclude that many factors may aid or prevent the metastatic process, that none of these can alone determine its outcome. Susceptibility to spontaneous cell-mediated cytotoxicity is but one of these factors.

Supported by grant from the Institut National de la Santé et de la Recherche Médicale. We thank Miss Valerie Chanoina and Marie-Laure Dhery for their valuable technical assistance throughout this study, and Miss Christine Labat for the secretarial assistance.

### References

BANIYASH, M., NETANEL, T. & WITZ, I.P. (1981). Differences in cell density associated with differences in lung colonizing ability of B16 melanoma cells. *Cancer Res.*, **41**, 433.

BECKER, S. & KLEIN, E. (1976). Decreased natural killer effect in tumor-bearing mice and its relation to immunity against oncornavirus-determined cell surface antigens. *Europ. J. Immunol.*, **6**, 892.

BROOKS, C.G., WAYNER, E.A., WEBB, P.J., DRAY, J.D., KENWRICK, S. & BALDWIN, R.W. (1981). The specificity of rat natural killer cells and cytotoxic macrophages on solid tumour derived target cell and selected variants. *J. Immunol.*, **127**, 2477.

BROOKS, C.F., FLANNERY, G.R., WILLMOTT, N., AUSTIN, E.B., KENWRICK, S. & BALDWIN, R.W. (1981). Tumour cells in metastatic deposits with altered sensitivity to natural killer cells. *Cancer Res.*, **28**, 191–198.

CIFONE, M.A., KRIPKE, M.L. & FIDLER, I.J. (1979). Growth rate and chromosome number of tumor cell lines with different metastatic potential. *J. Supramol. Str.*, **11**, 467.

CHEN, T.R. (1977). *In situ* detection of mycoplasma contamination in cell culture by fluorescent Hoechst 33258 stain. *Exp. Cell. Res.*, **104**, 255.

COLLINS, J.L., PATEK, P.O. & COHN, M. (1981). Tumorigenicity and lysis by natural killers. *J. Exp. Med.*, **153**, 89.

EHRLICH, R., EFRATI, M., BAR-EYAL, A. & 4 others. (1980). Natural cellular reactivities mediated by splenocytes from mice bearing three types of primary tumour. *Int. J. Cancer*, **26**, 315.

FIDLER, I.J., GRUYS, E., CIFONE, M.A., BARNES, Z. & BULANA, C. (1981). Demonstration of multiple phenotypic diversity in a murine melanoma of recent origin. *J. Natl Cancer Inst.*, **67**, 947.

GORELIK, E., SEGAL, S. & FELDMAN, M. (1979). Differences in resistance of metastatic tumor cells and cells for non local tumor growth to cytotoxicity of natural killer cells. *J. Natl Cancer Inst.*, **63**, 1397.

GIDLUND, M., ORN, A., PATTENGALE, P.K., JANSSON, P.K., WIGZELL, M. & NILSSON, K. (1980). Natural killer cells kill tumour cells at given stage of differentiation. *Nature* (*London*), **292**, 848.

HANNA, N. & FIDLER, I.J. (1981). Relationship between metastatic potential and resistance to natural killer cell mediated cytotoxity in three murine tumor systems. *J. Natl Cancer Inst.*, **66**, 1183.

HANNA, N. & BURTON, R.C. (1981). Definitive evidence that natural killer NK cells inhibit experimental tumour metastasis *in vivo*. *J. Immunol.*, **127**, 1754.

HEPPNER, G.H., DEXTER, D.L., DENUCCI, T., MILLER, F.R. & CALABRESI, P. (1978). Heterogeneity in drug sensitivity among tumor cell sub-populations of a single mammary tumor. *Cancer Res.*, **38**, 3758.

HILGARD, P. (1973). The role of blood platelets in experimental metastases, *Br. J. Cancer*, **28**, 429.

JONES, P.A. & DECLERCK, Y. (1980). Destruction of extracellular matrices containing glycoproteins, elastin and collagen by metastatic human tumor cells. *Cancer Res.*, **40**, 3222.

KARPATKIN, S. & PEARLSTEIN, E. (1981). Role of platelets in tumor cell metastases. *Annals of Internal Medicine*, **95**, 636.

LIOTTA, L.A., TRYGGVASON, K., GARBISA, S., HART, I., FOLTZ, C.M. & SHAFIE, S. (1980). Metastatic potential correlates with enzymatic degradation of basement membrane collagen. *Nature*, **284**, 67.

MANTOVANI, A., GIAVAZZI, R., ALESSANDRI, G., SPREAFICO, F. & GARATTINI, S. (1981). Characterization of tumor lines derived from spontaneous metastases of a transplanted murine sarcoma. *Eur. J. Cancer*, **17**, 71.

MONTAGNIER, L. (1966). La croissance sélective en gélose de cellules transformées par un virus cancérogène. *Pathol. Biol.*, **14**, 244.

NERI, A., RUOSLAHTI, E. & NICOLSON, G.L. (1981). Distribution of fibronectin on clonal cell lines of a rat mammary adenocarcinoma growing *in vitro* and *in vivo* at primary and metastatic sites. *Cancer Res.*, **41**, 5082.

NUNN, M.E., HERBERMAN, R.B. & HOLDEN, T. (1977). Natural mediated cytotoxicity in mice against non lymphoid tumor cells and normal cells. *Int. J. Cancer*, **20**, 381.

POSTE, G., DOLL, J., HART, I.R. & FIDLER, I.J. (1980). *In vitro* selection of murine B16 melanoma variants with enhanced tissue invasive properties. *Cancer Res.*, **40**, 1636.

POSTE, G. & FIDLER, I.J. (1980). The pathogenesis of cancer metastasis. *Nature*, **283**, 139.

PREHN, R.T. (1970). Analysis of antigenic heterogeneity within individual 3-methylcholanthrene induced mouse sarcoma. *J. Natl Cancer Inst.*, **45**, 1039.

RODER, J.C., KIESSLING, R., BIBERFELD, P. & ANDERSSON, B. (1978). Target-effector interaction in the natural killer (NK) cell system. II The isolation of NK cells and studies on the mechanism of killing. *J. Immunol.*, **121**, 2509.

SCHIRRMACHER, V., SHANTZ, G., CLAUER, K., KOMITOWSKI, D., ZIMMERMANN, H.P. & LOHMANN-MATTHES, M.L. (1979). Tumor metastases and cell-mediated immunity in a model system in DBA/2 mice. I: Tumor invasiveness *in vitro* and metastasis formation *in vivo*. *Int. J. Cancer*, **23**, 233.

SLOANE, B.F., HONN, K.V., SADLER, J.G., TURNER, W.A., KIMPSON, J.J. & TAYLOR, J.D. (1982). Cathepsin B activity in B16 melanoma cells: a possible marker for metastatic potential. *Cancer Res.*, **42**, 980.

STACKPOLE, C.W. (1981). Distinct lung-colonizing and lung metastasizing cell populations in B16 mouse melanoma. *Nature*, **289**, 798.

SWEENEY, F.L., POT-DEPRUN, J., POUPON, M.F. & CHOUROULINKOV, I. (1982). Heterogeneity of the growth and metastatic behaviour of cloned cell lines derived from a primary rhabdomyosarcoma. *Cancer Res.*, **43**, 3776.

TSURUO, T. & FIDLER, I.J. (1981). Differences in drug sensitivity among tumor cells from parental tumors selected variants and spontaneous metastases. *Cancer Res.*, **41**, 3058.

VLODAVSKY, I., SCHIRRMACHER, V., ARIAV, Y. & FUKS, Z. (1982). Lymphoma cell interaction with cultured vascular endothelial cells and with the subendothelial basal lamina: attachment, invasion and morphological appearance. (In press).

WANG, N., YU, S.H., LIENER, I.E., HEBBEL, R.P. EATON, J.W. & Mac KHANN, C.F. (1982). Characterization of high and low metastatic clones derived from a methylcholanthrene-induced murine fibrosarcoma. *Cancer Res.*, **42**, 1046.

WANG, B.S., McLOUGHLIN, G.A., RICHIE, J.P. & MANNICK, J.A. (1980). Correlation of the production of plasminogen activator with tumor metastasis in B16 mouse melanoma cell lines. *Cancer Res.*, **40**, 288.

YUNG, W.K.A., SHAPIRO, J.R. & SHAPIRO, W.R. (1982). Heterogeneous chemosensitivities of subpopulations of human glioma cells in culture. *Cancer Res.*, **42**, 992.